# Primary osteosarcoma of the scapula in an adolescent: a case report and review of the literature

**DOI:** 10.1097/RC9.0000000000001040

**Published:** 2026-09-23

**Authors:** Ibrahim Aljnide, Nawwar Shaheen, Rand A. Eid, Yasser Iskandar

**Affiliations:** a Faculty of Medicine, Damascus University, Damascus, Syrian Arab Republic; b Faculty of Medicine, Al-Andalus University for Medical Sciences, Damascus, Syrian Arab Republic; c Department of Surgery, Faculty of Medicine, Damascus University, Damascus, Syrian Arab Republic

**Keywords:** adolescent, case report, neoadjuvant chemotherapy, primary osteosarcoma of the scapula, total scapulectomy

## Abstract

**Background:**

Osteosarcoma is the most common primary malignant bone tumor, yet its occurrence in the scapula is exceptionally rare. The complex anatomy of the shoulder girdle and nonspecific presenting symptoms often lead to diagnostic delays.

**Case presentation:**

A 16-year-old male with no significant past medical history presented with several months of progressive swelling and pain in the right scapular region. Magnetic resonance imaging revealed a large, heterogeneous mass arising from the scapula with an extensive soft-tissue component. Core needle biopsy confirmed high-grade conventional osteoblastic osteosarcoma (Enneking stage IIB, AJCC stage IIB). Staging investigations, including chest computed tomography and a bone scan, revealed no evidence of metastatic disease. The patient received neoadjuvant chemotherapy following the Methotrexate, Adriamycin (doxorubicin), and Platinol (cisplatin) protocol (high-dose methotrexate, doxorubicin, and cisplatin). A subsequent radical en bloc total scapulectomy achieved negative margins (R0 resection). Postoperative histopathology demonstrated greater than 90% tumor necrosis (Huvos grade IV). No bony reconstruction was performed; a flail shoulder was accepted to avoid implant-related complications.

**Discussion:**

At 18 months postoperatively, the patient remains disease-free, with no evidence of local recurrence or metastasis. Functional assessment using the Musculoskeletal Tumor Society score yielded 63%, and the Toronto Extremity Salvage Score was 68%. The patient has completed all adjuvant chemotherapy cycles without significant complications.

**Conclusion:**

This case demonstrates that multidisciplinary management – neoadjuvant chemotherapy followed by radical resection with negative margins – can achieve favorable local control. While total scapulectomy sacrifices shoulder abduction, it preserves hand and elbow function, offering a viable limb-sparing option. The rarity of this tumor underscores the importance of continued case reporting to refine diagnostic and therapeutic approaches.

## Introduction

Osteosarcoma, the most common primary malignant bone tumor, has an annual incidence of 3–4 per million and accounts for ~20% of primary bone sarcomas^[^1^]^. It typically arises in long bone metaphyses (e.g., the knee and proximal humerus) during adolescent growth spurts^[^2^]^, whereas flat bone involvement (the scapula, pelvis, and skull) is rare (<5%)^[^3^]^. Primary scapular osteosarcoma is exceptionally rare, with only sporadic reports^[^4–7^]^. Its atypical location often delays diagnosis^[^8^]^, and the complex anatomy – close to the chest wall and neurovascular structures (the axillary vessels and brachial plexus) – poses unique surgical challenges^[^9^]^. Clinically, it presents with insidious shoulder pain and a growing mass, mimicking rotator cuff pathology, bursitis, or benign tumors, which delays diagnosis^[^10^]^. Imaging [radiography, computed tomography (CT), and magnetic resonance imaging (MRI)] is crucial to reveal aggressive features such as cortical destruction, soft tissue extension, and matrix mineralization^[^11^]^. Prognosis for osteosarcoma has improved with neoadjuvant chemotherapy (5-year survival ~70–80% for localized disease)^[^12^]^, but scapular outcomes are less well defined. Negative margins, histologic response, tumor size, and metastases are key prognostic factors^[^13^]^. This SCARE-compliant case report^[^14^]^ presents a rare primary high-grade osteoblastic scapular osteosarcoma in an adolescent, detailing the diagnostic workup, multidisciplinary management, surgical strategy, and medium-term outcomes, alongside a literature review focused on adolescent cases.

HIGHLIGHTS

- This case emphasizes the critical importance of considering primary osteosarcoma in the differential diagnosis of a progressively enlarging, painful scapular mass in an adolescent, given the nonspecific presentation and potential for diagnostic delay.
- Achieving a good histological response (Huvos grade III–IV, > 90% tumor necrosis) following neoadjuvant Methotrexate, Adriamycin (doxorubicin), and Platinol (cisplatin) chemotherapy serves as a strong prognostic indicator in scapular osteosarcoma, even when radiographic response is limited.
- Radical en bloc total scapulectomy with negative margins (R0 resection) is the cornerstone of local control, and forgoing bony reconstruction – accepting a flail shoulder – can avoid implant-related complications while preserving hand and elbow function.

## Case presentation

A 16-year-old right-hand-dominant male with no significant past medical or family history and no prior trauma or radiation exposure presented with a large swelling over his right scapular region. The mass had been gradually increasing in size over several months. Initially associated with mild discomfort, the pain became progressively more severe, eventually leading to functional limitation of his right shoulder movement. His body mass index was 22.4 kg/m^2^, and his Eastern Cooperative Oncology Group performance status was 1.

On physical examination, a massive, dome-shaped mass was observed on the patient’s right upper back (Fig. 1). The overlying skin was stretched, with visible striae, and the mass itself was firm to hard on palpation, with deep fixation, suggesting origin from the underlying bone. The mass measured approximately 18 cm in greatest diameter. There was no palpable lymphadenopathy. Range of motion of the right shoulder was significantly limited, with active forward flexion to 90°, abduction to 70°, and external rotation to 30°, limited by pain and mechanical obstruction. The neurovascular examination of the right upper extremity was normal, with intact motor function and sensation.

**Figure 1.**
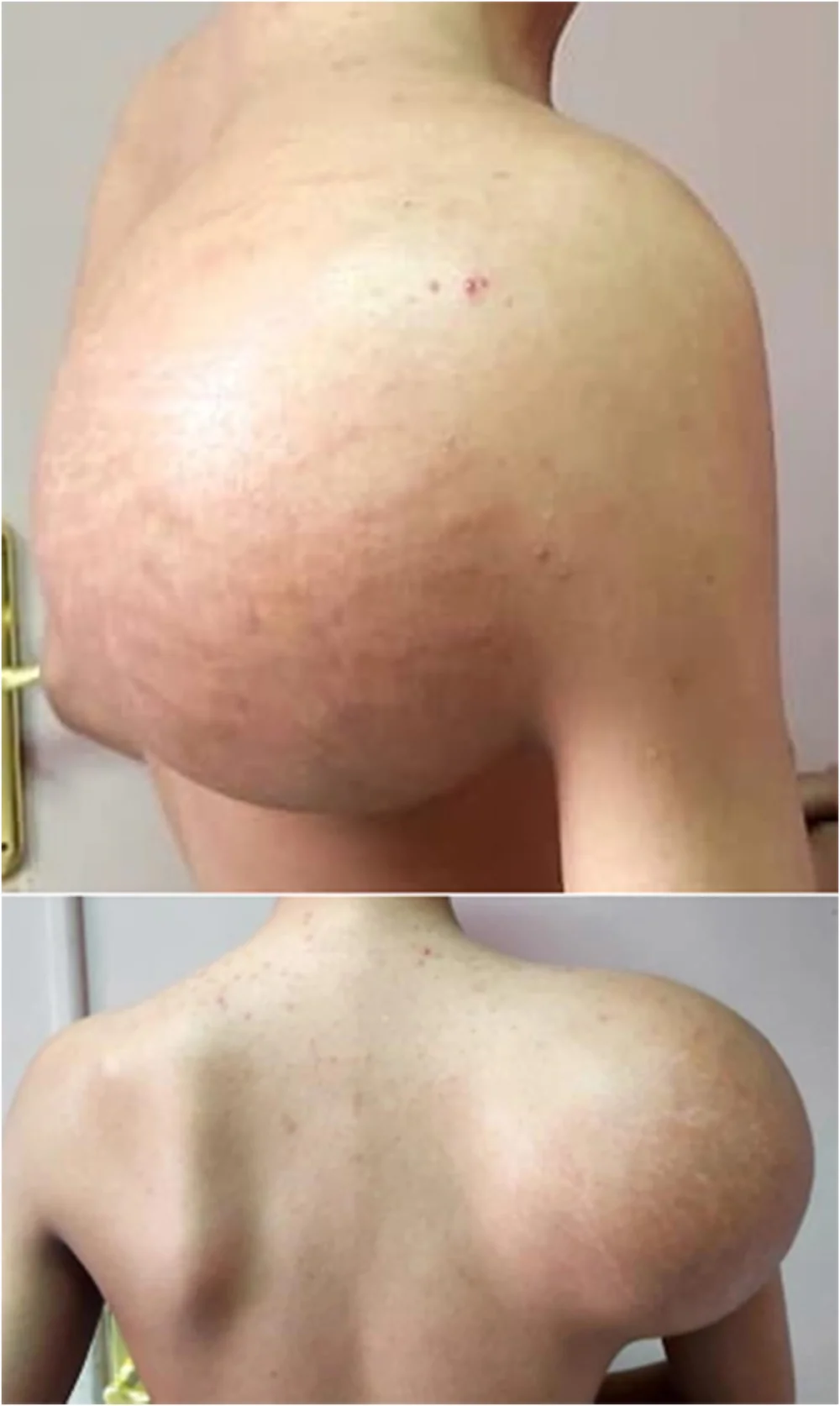
Clinical photograph of the right scapular region demonstrating a large, dome-shaped mass causing marked asymmetry of the right shoulder. The overlying skin is stretched with visible striae. The mass measures approximately 18 cm in its greatest diameter. Photograph obtained in posterior view.

Laboratory studies revealed an elevated serum alkaline phosphatase (ALP) level of 425 U/L (normal, 40–150 U/L) and a lactate dehydrogenase (LDH) level of 320 U/L (normal, 100–200 U/L). Complete blood count and comprehensive metabolic panel were within normal limits.

MRI of the right shoulder revealed a large, heterogeneous mass arising from the right scapula (Fig. 2). The lesion measured 17.5 × 12.0 × 9.5 cm and demonstrated mixed signal intensity on both T1-weighted (intermediate to low signal) and T2-weighted (heterogeneously hyperintense) sequences, consistent with necrosis, hemorrhage, and tumor matrix. The mass showed extensive soft tissue extension, encasing the infraspinatus and subscapularis muscles, though without clear invasion of the thoracic cavity (Fig. 2B, axial T2-weighted image). Post-contrast sequences demonstrated heterogeneous enhancement, with areas of necrosis. The lesion did not involve the glenohumeral joint.

**Figure 2.**
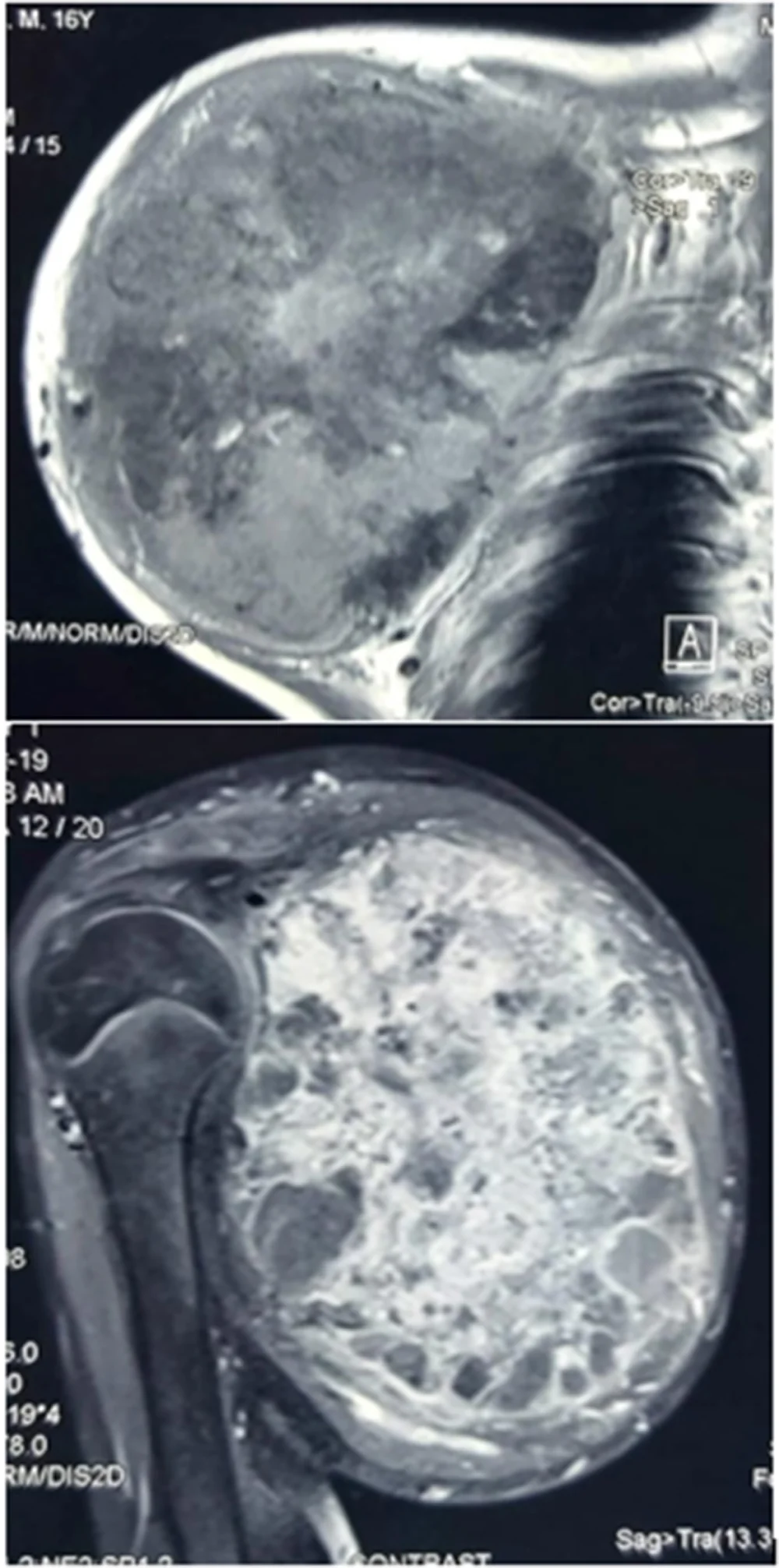
Magnetic resonance imaging of the right scapula. (A) Coronal T1-weighted image (TR/TE: 600/15 ms) showing a large, heterogeneous mass arising from the right scapula with intermediate-to-low signal intensity, measuring 17.5 × 12.0 × 9.5 cm. The mass demonstrates extensive involvement of the scapula, with soft-tissue extension. (B) Axial T2-weighted image with fat suppression (TR/TE: 3500/85 ms) demonstrating the mass with heterogeneously hyperintense signal, consistent with necrosis, hemorrhage, and tumor matrix. The lesion shows extensive soft-tissue extension without clear invasion of the thoracic cavity. The relationship to the axillary vessels and brachial plexus is demonstrated.

Staging investigations included a contrast-enhanced CT of the chest, which demonstrated no pulmonary metastases. A whole-body technetium-99 m methylene diphosphonate bone scan revealed intense radiotracer uptake localized to the right scapular region, with no evidence of distant osseous metastases.

A subsequent core needle biopsy of the mass yielded histopathology showing a highly cellular malignant mesenchymal tumor composed of pleomorphic spindle and polygonal cells. The cells contained hyperchromatic nuclei with high mitotic activity (15 mitoses per 10 high-power fields), and malignant osteoid deposition was present, leading to a diagnosis of high-grade osteoblastic osteosarcoma. Immunohistochemistry was performed and showed diffuse positivity for SATB2 and osteocalcin, with negative staining for cytokeratin, EMA, and S-100, excluding alternative diagnoses.

The patient received neoadjuvant chemotherapy following the Methotrexate, Adriamycin (doxorubicin), and Platinol (cisplatin) (MAP) protocol, which included high-dose methotrexate (10 g/m^2^ IV) with leucovorin rescue, doxorubicin (75 mg/m^2^ IV), and cisplatin (120 mg/m^2^ IV). Three preoperative cycles were administered at 3-week intervals. The patient tolerated chemotherapy reasonably well; no dose reductions were required.

Imaging following neoadjuvant therapy showed only a mild reduction in tumor vascularity, with no significant decrease in tumor size.

Following neoadjuvant chemotherapy, the patient underwent a radical en bloc resection with total scapulectomy. The surgical procedure was performed with the patient in the lateral decubitus position. An elliptical incision was made over the tumor, encompassing the previous biopsy tract. Wide soft tissue dissection was performed to achieve adequate margins, with a planned 2-cm margin of normal tissue. The axillary vessels and brachial plexus were identified and preserved (Fig. 3). The subclavian vessels were carefully dissected and protected.

**Figure 3.**
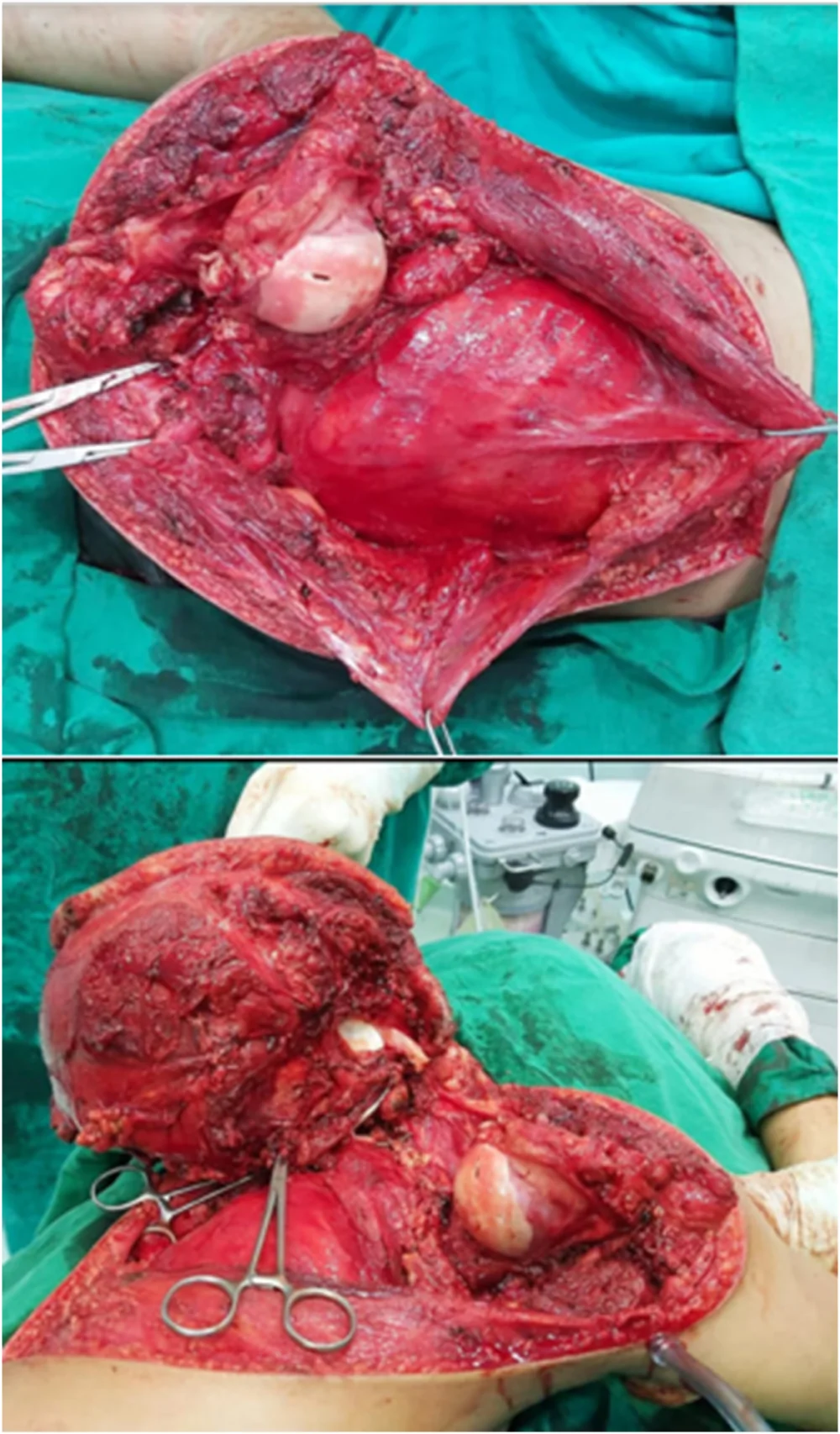
Intraoperative photographs during total scapulectomy. The patient is in the lateral decubitus position with an elliptical incision over the tumor. The axillary vessels and brachial plexus have been identified and preserved (indicated by arrows). The scapula is being mobilized by detaching its muscular attachments. The use of vessel loops facilitates neurovascular dissection and protection. (A) Pre-resection view showing the tumor and surrounding tissues. (B) Post-resection view showing the tumor bed with preserved neurovascular structures.

The surgical approach involved the following:

- **Muscles sacrificed**: trapezius (lower portion), infraspinatus, subscapularis, teres major, teres minor, and latissimus dorsi (partial).
- **Muscles preserved**: deltoid (partially) and trapezius (upper portion).
- **Glenoid**: Resected (total scapulectomy).
- **Clavicle**: preserved.

- **Neurovascular structures**: axillary artery, axillary vein, and brachial plexus (all preserved with clear margins).

The tumor was found to be massive, replacing the entire scapula with extensive involvement of the surrounding muscles. No gross invasion of the chest wall or the neurovascular bundle was identified. After detaching all muscular attachments, the entire scapula with the tumor was removed en bloc. No bony reconstruction was performed, with a flail shoulder accepted to avoid implant-related complications, in accordance with the patient’s and family’s preferences after a thorough discussion of the risks and benefits.

Gross examination of the postoperative specimen revealed a large, lobulated mass replacing the scapula, measuring 18.5 × 13.0 × 10.0 cm, with a variegated cut surface showing gray-white tumor areas, hemorrhage, and necrosis. The tumor extended into the surrounding soft tissues, with a maximum soft-tissue extension of 4.5 cm.

Microscopic examination confirmed a high-grade malignant spindle cell tumor with marked cellular pleomorphism, frequent atypical mitoses, abundant malignant osteoid formation, and areas of chondroid differentiation.

All surgical margins were negative for tumor involvement.

The tumor showed no vascular invasion, no perineural invasion, and no lymphovascular invasion. The pathological stage was pT2bN0M0 (AJCC stage IIB). The final diagnosis was high-grade osteoblastic osteosarcoma with a good histological response to chemotherapy (Huvos grade IV, greater than 90% necrosis).

The patient’s postoperative recovery was uneventful. The surgical wound healed primarily, and the patient was discharged on postoperative day 5. He was fitted with a shoulder sling for comfort and instructed on passive range-of-motion exercises for the elbow and hand. No adjuvant radiotherapy was administered.

The patient completed adjuvant chemotherapy, receiving three additional cycles of the MAP protocol (three cycles preoperatively and three cycles postoperatively, for a total of six cycles). The patient tolerated adjuvant chemotherapy well, with no dose delays or significant complications.

At 18 months, the patient remains disease-free on surveillance MRI (6, 12, and 18 mo) and chest CT (3, 6, 12, and 18 mo), with normalized ALP (85 U/L) and LDH (145 U/L). Functional outcomes: Musculoskeletal Tumor Society (MSTS) 63% (19/30), Toronto Extremity Salvage Score (TESS) 68%; active shoulder forward flexion 45°, abduction 30°, external rotation 15°, internal rotation to L5; normal hand and elbow function; VAS pain score 2/10 (mild, intermittent). He is independent in daily activities (using compensatory strategies for overhead tasks), wears a shoulder orthosis for prolonged activities, and reports satisfaction with return to studies and social life.

## Discussion

Primary osteosarcoma of the scapula is an exceptionally rare malignancy, accounting for less than 1% of all osteosarcomas^[^3^]^. This case of a 16-year-old male with a high-grade conventional osteoblastic osteosarcoma of the scapula illustrates the diagnostic challenges, multidisciplinary management, and critical prognostic factors associated with this uncommon location. The rarity of this presentation is reflected in the limited literature, with only sporadic case reports and small series describing this entity^[^4–7,15^]^.

Comparison with Previously Reported Cases in Table 1. A review of the literature reveals fewer than 50 reported cases of primary scapular osteosarcoma. The mean age at presentation is approximately 38 years (range: 12–78 years), with a slight male predominance. The most common histological subtype is conventional osteoblastic osteosarcoma, as in our case, though rare variants including telangiectatic, parosteal, periosteal, and epithelioid subtypes have been described^[^6,7,16^]^.

**Table 1:** Summary of previously reported adolescent scapular osteosarcoma cases.

Author/year	Age/sex	Histology	Size (cm)	Necrosis	Margins	Reconstruction	Follow-up	Outcome
Present case	16/M	Osteoblastic	18.5	>90% (Huvos IV)	R0	None	18 mo	NED
Choudhary *et al* 2008^[^5^]^	14/M	Osteoblastic	NR	NR	R0	Allograft	24 mo	NED
Herget *et al* 2014^[^6^]^	15/F	Epithelioid	NR	NR	R0	None	36 mo	NED
Garayua-Cruz *et al* 2025^[^15^]^	17/M	Osteoblastic	NR	>90%	R0	Implant	48 mo	NED
Hong *et al* 2014^[^7^]^	16/M	Periosteal	8.0	N/A	R0	None	12 mo	NED

NED, no evidence of disease; NR, not reported; mo, months; N/A, not applicable (periosteal osteosarcoma does not receive neoadjuvant chemotherapy).

Our case is notable for several features: (1) the exceptionally large tumor size (18.5 cm) in an adolescent, (2) the excellent histological response (>90% necrosis) to MAP chemotherapy, (3) the achievement of R0 resection despite the large tumor and complex anatomy, and (4) the acceptance of a flail shoulder without reconstruction, which, to our knowledge, represents a conscious decision that has been reported in only a few cases.

The diagnosis of scapular osteosarcoma is often delayed due to nonspecific symptoms and rarity^[^8^]^; our patient’s several-month delay aligns with literature^[^5,15^]^. Differential diagnoses include primary bone tumors, metastases, and benign entities like aneurysmal bone cysts, fibrous dysplasia, or chondrosarcoma^[^10^]^. MRI is critical for delineating tumor extent, neurovascular involvement, and chest wall invasion and was instrumental in surgical planning^[^17^]^. Staging (chest CT for pulmonary metastases, bone scintigraphy/PET-CT for osseous spread^[^18^]^) confirmed localized stage IIB disease. Negative surgical margins (R0) are critical, as local recurrence worsens survival^[^19^]^; in a Mayo series, recurrence (all within 2 years) significantly reduced disease-specific survival (HR 13.19)^[^15^]^. Histological response (Huvos grading) is prognostic, with grade IV (>90% necrosis) linked to >80% 5-year survival^[^20,21^]^; our patient achieved Huvos IV. Tumor size greater than 15 cm is generally adverse^[^22^]^, yet our 18.5 cm tumor responded excellently, remaining disease-free at 18 months. Surgical goals are R0 resection and maximal functional preservation^[^9,23^]^. Given extensive involvement, we performed total scapulectomy, accepting a flail shoulder with expected loss of abduction and flexion but preserved hand and elbow function. Reconstruction (autograft, allograft, or endoprosthetic^[^24^]^) is debated; we opted against bony reconstruction to minimize implant-related complications^[^15^]^, operative time, and blood loss, aligning with evidence showing no clear survival or functional advantage^[^24,25^]^. Functional outcomes (MSTS 63%, TESS 68%, forward flexion 45°, abduction 30° at 18 months) align with literature^[^15,26^]^; preserved hand and elbow function enabled daily activities, and the patient is satisfied. MAP chemotherapy remains standard^[^27^]^. We administered three well-tolerated preoperative cycles^[^28^]^ (achieving Huvos IV despite a large or flat-bone location^[^29^]^) followed by adjuvant cycles to target micrometastases^[^30^]^.

## Limitations

However, we acknowledge several limitations. First, this is a single case report, and our findings may not be generalizable. Second, although the 18-month follow-up is informative, longer follow-up is needed to fully assess the risk of late recurrence. Third, the functional assessment is limited to a single time point; serial assessments would provide a more complete picture of functional recovery and adaptation over time.

## Conclusion

Primary osteosarcoma of the scapula is an exceptionally rare malignancy that presents diagnostic and therapeutic challenges due to the complex anatomy of the shoulder girdle. This case of a 16-year-old male with high-grade conventional osteoblastic osteosarcoma demonstrates that multidisciplinary management – including neoadjuvant chemotherapy, radical resection with negative margins, and objective assessment of histological response – can achieve favorable local control. Total scapulectomy predictably results in permanent loss of shoulder abduction but preserves hand and elbow function, achieving satisfactory functional outcomes without the need for reconstruction. At the 18-month follow-up, our patient remains disease-free with acceptable functional status, though longer follow-up is necessary to confirm durable disease control. The rarity of this tumor underscores the value of continued case reporting to refine diagnostic approaches, surgical strategies, and our understanding of long-term outcomes.

## Data Availability

The data that support the findings of this study are available from the corresponding author upon reasonable request.

## Author’s statement

None of the authors listed in the manuscript are employed by a government agency that has a primary function other than research and/or education. Moreover, none of the authors are submitting this manuscript as an official representative or on behalf of the government.
